# PocketMatch: A new algorithm to compare binding sites in protein structures

**DOI:** 10.1186/1471-2105-9-543

**Published:** 2008-12-17

**Authors:** Kalidas Yeturu, Nagasuma Chandra

**Affiliations:** 1 Bioinformatics Centre and Supercomputer Education and Research Centre, Indian Institute of Science, Bangalore – 560 012, India

## Abstract

**Background:**

Recognizing similarities and deriving relationships among protein molecules is a fundamental requirement in present-day biology. Similarities can be present at various levels which can be detected through comparison of protein sequences or their structural folds. In some cases similarities obscure at these levels could be present merely in the substructures at their binding sites. Inferring functional similarities between protein molecules by comparing their binding sites is still largely exploratory and not as yet a routine protocol. One of the main reasons for this is the limitation in the choice of appropriate analytical tools that can compare binding sites with high sensitivity. To benefit from the enormous amount of structural data that is being rapidly accumulated, it is essential to have high throughput tools that enable large scale binding site comparison.

**Results:**

Here we present a new algorithm *PocketMatch *for comparison of binding sites in a frame invariant manner. Each binding site is represented by 90 lists of sorted distances capturing shape and chemical nature of the site. The sorted arrays are then aligned using an incremental alignment method and scored to obtain PM*Scores *for pairs of sites. A comprehensive sensitivity analysis and an extensive validation of the algorithm have been carried out. A comparison with other site matching algorithms is also presented. Perturbation studies where the geometry of a given site was retained but the residue types were changed randomly, indicated that chance similarities were virtually non-existent. Our analysis also demonstrates that shape information alone is insufficient to discriminate between diverse binding sites, unless combined with chemical nature of amino acids.

**Conclusion:**

A new algorithm has been developed to compare binding sites in accurate, efficient and high-throughput manner. Though the representation used is conceptually simplistic, we demonstrate that along with the new alignment strategy used, it is sufficient to enable binding comparison with high sensitivity. Novel methodology has also been presented for validating the algorithm for accuracy and sensitivity with respect to geometry and chemical nature of the site. The method is also fast and takes about 1/250^*th *^second for one comparison on a single processor. A parallel version on BlueGene has also been implemented.

## Background

Much of present day biology is dependent on sequence-structure-function relationships in protein molecules, insights obtained for one protein heavily influencing understanding of other proteins in the family. Recognizing similarities and deriving relationships therefore is a fundamental objective in bioinformatics. Some of these similarities are obvious at the sequence level while some are detected at the structure level [1,2]. It is in fact well established now that the conservation at the structure level of related proteins can be higher and hence much more detectable than at the sequence level [3]. In this context, there are a number of examples in the literature, which illustrate that structures often convey the 'meaning', more efficiently than sequences, here 'meaning' referring to the 'function' of the protein. On the other hand, there are also a number of instances, which illustrate that a particular 'function' is achieved by proteins whose sequences and structures are dis-similar. For example, at least three different proteins with different folds and architectures recognize mannose and exhibit mannose-mediated physiology [4]. In other words, structures also fail to convey the 'meaning' in many cases. We do not yet know if this failure is because of our inability to recognize any similarities in such seemingly dis-similar proteins or it is simply because no similarities actually exist among them. What ultimately matters for a protein molecule however, is its function and not what means it uses to achieve it [5]. A given function could be conserved simply by having similarities in some elements of the structure, such as the binding site residues [6]. A classic example is the large family of serine proteases which are classified into different sequence and structural families, but all come under the functional class of serine proteases due to the presence of the catalytic triad [7]. Comparison of binding sites differ from comparison of whole structures for two main reasons (a) the binding sites are small containing only a few residues and (b) these residues are often not contiguous in sequence. Alignment of two sites containing discrete sets of atoms involves evaluation of a huge number of mappings. This makes it important to have efficient algorithms with low time and space complexities that are capable of identifying and ranking different extents of similarities appropriately. With several structural genomics projects as well as advances in computational methods for structure prediction, the structural databases are growing at a rapid pace, providing experimental structures of thousands and confident homology models of millions of protein molecules [8,9]. Algorithms for identification of binding pockets with reasonable confidence have also been developed [10-13]. The need for large scale comparisons of binding sites is hence accentuated. Some methods are already available for such a purpose based on ideas well established in the field of image processing. For example SitesBase [14] that uses 'Geometric hashing' involves selection of triads of points representing atomic types and positions in each site and comparing the triangles formed by triads; PINTS [15] uses a depth first traversal strategy, adopted to find common set of nodes between a pair of graphs connected by similar pattern of edges; Spherical harmonics based algorithm [16], captures distribution of points representing the site in terms of coefficients of a square integrable function on a unit sphere; CavBase [17] identifies maximal common sub-graphs between pairs of sites; SPASM and RIGOR [18] compares distribution of residues from the centroid of the binding sites. Each of the methods have their own merits and demerits, warranting exploration of newer methods for site comparison. Here we present a new algorithm called *PocketMatch *for representing a binding site in a frame invariant manner and comparison of pairs of sites based on alignment of sorted sequences of distances between pairs of points representing sites.

## Results and discussion

A new efficient and accurate algorithm *PocketMatch *has been developed for comparing binding sites in protein structures. A comparison between a pair of sites of an average size of 50 atoms takes only 1/250^*th *^of a second on a single processor. The algorithm has been used for large scale database searches and all-vs-all comparisons. A typical database search of a query site against those from a large dataset comprising about 20000 sites takes in the order of 4 minutes to complete on the same machine with MPI-C version using 4 processors.

### Algorithm

Comparison of a pair of binding sites involves three aspects, (a) representation of each site as sorted lists of distances between chosen points, (b) alignment of two sets of distance lists and (c) choosing a scoring scheme for arriving at a final score. Our representation scheme for the site is based on capturing the geometry of a 3*D *object in a 1*D *representation by a set of all pairs of distances between points. Such a set of distances would become a frame-invariant representation, a highly desirable scheme for any general shape comparison method. Two sets of distances can be compared for similarity by considering a suitable mapping between distances whose dissimilarity is bounded by a small amount. As the number of such mappings of distances from one set to the other would be huge, we represent them as sorted sequences in ascending order. An alignment strategy for comparison of two sequences of distances is also presented. The different steps involved are described.

### Representation of binding site

**Step-1 **A set of residues whose one or more atoms surround a given crystallographic ligand within a specified distance (4Å as default) from each atom of the ligand is taken as the binding site. We chose to consider atoms of the complete residues corresponding to each of these atoms for shape-representation. For any 3*D *object represented by a set of points, the set of all pairs of distances between the points encodes the shape of the object. These points can then be flagged with specific chemical properties. 20 amino acids were considered in 5 groups – Group-0:(A,V,I,L,G,P); Group-1:(K,R,H); Group-2:(D,E,Q,N); Group-3:(Y,F,W); Group-4:(C,S,T).

Grouping is implemented as a user defined parameter enabling use of other types of grouping.

**Step-2 **Represent each complete residue of the site by 3 types of points – *C*_*α*_, *C*_*β *_and *C*_*centroid *_corresponding to C-Alpha, C-Beta and centroid of atoms of the side chain of the residue. Centroid is computed as

$$\bar{x}=\sum_{i=1}^{n}{\bar{x}}_{i}$$

where ${\bar{x}}_{i}$ indicates coordinates of the *i*^*th *^atom of the side chain and *n *is the number of atoms in the side chain.

**Step-3 **Binning of distances and representation format.

**(a) **Compute distances between all pairs of points and bin the distances into 90 sets corresponding to group-type-pair and point-type-pairs.

**(i) **There are 5 types of residue-groups. Therefore 5 * (5 - 1)/2 + 5 → 15 pairs of groups are possible.

**(ii) **There are 3 types of points. Therefore 3 * (3 - 1)/2 + 3 → 6 pairs of point types are possible.

**(iii) **Considering both residue-group and point-type information, total number of possible sets of distances would be 15 * 6 ⇒ 90.

**(b) **Write the above representation in the following format to a file.

$$\begin{array}{l} NGP\\ NTP\\ \left\{\begin{array}{lllll} ND_{1} & & & & \\ d_{1}, & d_{2}, & \cdots & d_{j}, & \cdots \end{array}\right\}\\ \left\{\begin{array}{lllll} ND_{2} & & & & \\ d_{1}, & d_{2}, & \cdots & d_{j} & \cdots \end{array}\right\}\\ \vdots \\ \left\{\begin{array}{lllll} ND_{90} & & & & \\ d_{1}, & d_{2}, & \cdots & d_{j} & \cdots \end{array}\right\} \end{array}$$

Where, NGP: Number of pairs of group-types, NTP: Number of pairs to point-types, *ND*_*i*_: Number of distances in the *i*^*th *^bin, *d*_*j*_: distance between *j*^*th *^pair of points.

**Step-4 **The distances in each list were sorted in ascending order.

### Alignment of a pair of distance-sets

To compute similarity score between two binding sites, each of the 90 sets of one site has to be compared with its corresponding set of the other site. In the next step, similarity between sets was computed using a *greedy strategy *for alignment of sorted distance-sequences.

**Step-5**: Let the given pair of sorted(ascending) distance lists be *S*_1 _and *S*_2 _where each element is indexed by *S*[*i*] for the list, *S*. Let the threshold for alignment of two distances be *τ *i.e., |*S*_1_[*i*] - *S*_2_[*j*]| ≤ *τ*. Let *m *= |*S*_1_| and *n *= |*S*_2_| denote the cardinalities of the sets *S*_1 _and *S*_2 _respectively. Let the variable *counter *hold the number of matched distances between two sequences. Then alignment strategy is as indicated in the (*Sub-routine 1*) below.

*Sub-routine *1 Alignment of a pair of sorted distance sequences

i = 0; j = 0; counter = 0;

**while **(*i *≤ *m*) ∧ (*j *≤ *n*) **do**

   **if **|*S*_1_[*i*] - *S*_2_[*j*]| ≤ *τ ***then**

      *i *← *i *+ 1; *j *← *j *+ 1

      *counter *← *counter *+ 1

   **else**

      **if ***S*_1_[*i*] <*S*_2_[*j*] **then**

         *i *← *i *+ 1;

      **else**

         *j *← *j *+ 1;

      **end if**

   **end if**

end while

### Scoring for similarity between sites

**Step-6: Scoring of the alignment**: An alignment score between a pair of sites is the net average of the number of matching distance elements in the 90 lists as a fraction of total number of distance elements in the bigger set, for a chosen threshold *τ*, as shown

$$PMScore=\frac{\sum_{i=1}^{90}Count_{i}}{maximum(|S_{1}|,|S_{2}|)}$$

where |*S*| indicates cardinality of the set.

This measure of similarity, referred to as PM*Score *is used as the default scoring scheme in this study. A variant of this scoring scheme PM*Score*_*min*_, in which the denominator was taken as the *minimum *(|*S*_1_|, |*S*_2_|), was also explored, since it emphasises local structural similarity ignoring the relative size mismatch between the two sites being compared. However we observed that the comparison was too insensitive with such a scheme and did not use it further. *τ *is an important parameter that governs the alignment, which decides when a given pair of distances constitute a match. Different values of *τ *(please see section on sensitivity analysis) were tried and a default of 0.5Å was used in this study.

### Testing

*PocketMatch *has been validated for two aspects, (a) first to validate how well known similarities are being reproduced and (b) how sensitive is the algorithm with respect to minor perturbations in geometry and residue types at the site. Comparison with known binding sites that are similar to each other is achieved by three ways (i) by considering proteins belonging to the same SCOP classification up to the family level, since such proteins are likely to have similar binding sites, given high similarity in their overall folds and their inferred homology within a family (ii) by comparing sites for the same ligand of multiple subunits in the same protein in a dataset of tetrameric proteins and finally (iii) by performing an all-vs-all comparison in a curated set of 51 sites for 4 ligand types corresponding to 27 proteins described in Table 1 and testing if the sites for each of the ligands cluster separately.

**Table 1:** Different datasets used in the study.

**No**.	**Dataset**	**Initial Size**	**Remarks**	**Final set used in the study**	
				
				Proteins	Sites
**i**	PDBBind	1091	One representative ligand of each type is considered in each protein; filtered to remove sites for small and covalently bound ligands; retained proteins common with the SCOP database	786	893

**ii**	PDBBind	1091	Filtered to remove sites for small, covalently bound ligand and all suggested by Jackson and co-workers (10) to contribute to noise; considered sites for all ligand types for the remaining	456	1146

**iii**	Curated dataset of CIT,MTX,MK1 and PGA	27	Ligands for varying sizes and types from PDBBind with multiple sites for a ligand in different proteins	27	51

**iv**	Tetramer	3768	Multiple sites for each ligand in the same protein; filtered to remove small and covalently bound ligands	1525	11301

#### Based on SCOP classification

**Figure 1:**
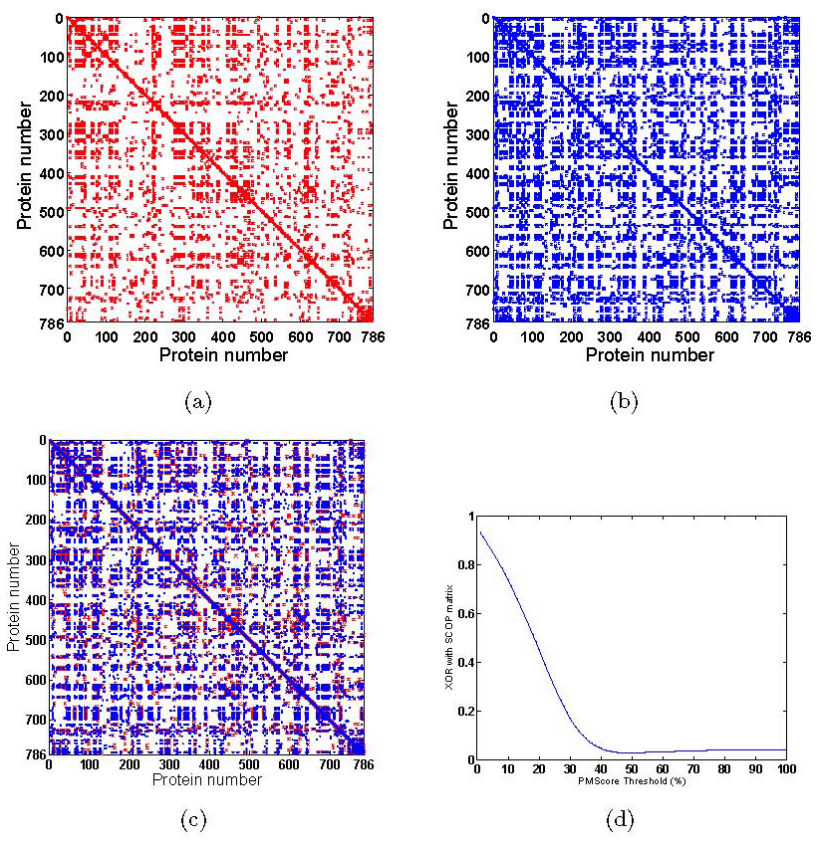
**Validation with respect to SCOP**. Validation of *PocketMatch *against SCOP. Visualization of (a) the PMS (M1) matrix and (b) the SCOP matrix (M2). Both are square matrices corresponding to 786 proteins illustrating results of all-pair comparisons. In both these a dot indicates the presence of similarity between the given protein-pair and a blank space indicates dis-similarity under the parameters defined (c) Superposition of PM*Score *(red) and SCOP(blue) matrices using XOR as described in the text. PM*Score *matrix was computed for 50%-threshold. Corresponding dis-similarity(XOR) and similarity scores are 0.0284 and 0.9716 (d) Plot showing the XOR values for different PM*Score *matrices varying in threshold from 0 to 100 percent.

399171 all-pair-site comparisons in PDBBind (Table 1-i) was carried out. Two matrices M1 and M2 were constructed as described in the Methods section. The first matrix shows similarities among the 786 proteins in the dataset used in the study based on the PocketMatch scores while the second matrix shows similarities in the same dataset based on similarities in the SCOP families they belong to. The first matrix thus estimates similarities in the binding sites of the proteins, while the second considers similarities in the overall fold, as illustrated in Figures 1(a) and 1(b). Two proteins that have sites scoring 50% or higher with *PocketMatch *are indicated by red dots, while those that do not have that level of similarity are again left as blank spaces. Those proteins which belong to the same SCOP family are indicated by blue dots, while blank spaces correspond to those that have different SCOP families, as shown in Figure 1(b). Given two matrices *M*_1 _and *M*_2 _– one for PM*Scores *and one for SCOP respectively, the dis-similarity between them has been calculated as *XOR*,

$$XOR=\frac{\sum_{\forall (i,j)}M_{1_{ij}}!=M_{2_{ij}}}{N^{2}}$$

where *N *is the dimension of the matrix.

Figure 1(c) indicates superposition to show similarities or XOR (and automatically also dis-similarities) of two matrices of the same dimension. The red and blue dots in the 2 matrices would superpose well only for protein pairs showing both SCOP family as well as binding site similarity. Where SCOP similarity is 0 and PM*Score *is also 0, a blank space will be present in both and hence will remain as blank space in the superposition also. Figure 1(c) illustrates superposed matrices, at a threshold of 50% for which the dis-similarity and similarity scores are 0.0284 and 0.9716 respectively.

*PocketMatch *matrices can be constructed at different thresholds, and the above excerise of superposition and computing XOR repeated for each threshold. Figure 1(d) illustrates XOR between the PM*Score *and the SCOP matrices as a function of PM*Score *threshold. As expected the XOR values start at a value close to 1 when the threshold is very low (at 0%) and inch towards 0 when the thresholds are increased to 100%. A sharp decline in XOR can be seen at around a threshold of 40%. This means that when a pair of sites match with a score of 40% or higher, they exhibit the same SCOP classification. At lower thresholds, for example at 20%, i.e., when two sites are considered similar to each other even when they match to only 20%, the correspondence of such a metric with the SCOP matrix is poor. This analysis serves to validate two points, (a) that higher PM*Scores *are reflective of true-positives in the context of the family level SCOP classification and (b) that lower PM*Scores *are reflective of true-negatives by the same logic. The graph also shows that a score of 40% is discriminatory enough to identify a true-positive.

#### Similarities in tetramers

**Figure 2:**
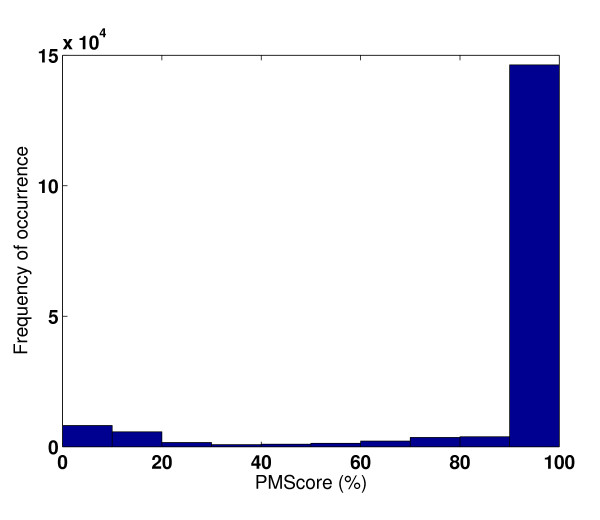
**Validation with respect to tetramers**. Histogram showing PM*Scores *in the tetramer dataset.

Next, we tested the *PocketMatch *algorithm by comparing multiple binding sites of the same ligand in the same protein. The ligand sites in the different subunits in the tetramer dataset (Table 1-iv) were compared. In all 174, 372 comparisons were carried out for the 11301 sites in 1525 tetramers. In most subunits, binding sites match with sites of other subunits of the same protein with a score of 90% to 100% (Figure 2). The two validation tests demonstrate high prediction accuracies, both in terms of assigning high scores to similar sites and low scores to dis-similar sites.

#### Using a curated dataset of known similarities

**Figure 3:**
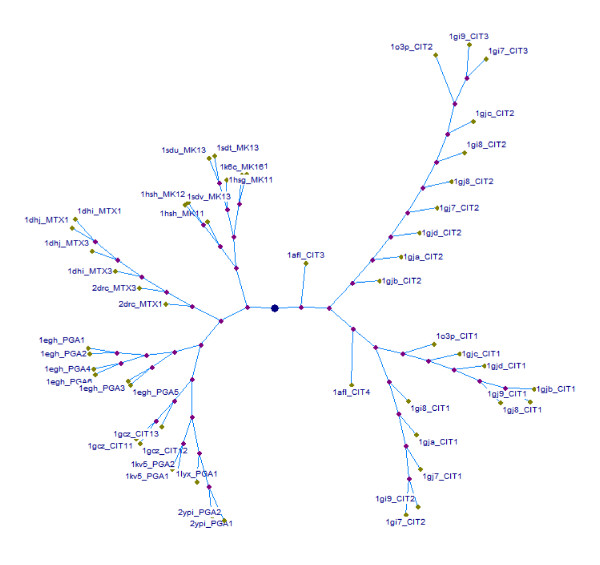
**Validation with respect to selected set of ligands**. Cluster-tree showing similarities in an all vs all comparison of 51 sites for four ligands in dataset-iii (Table 1). The nodes are labelled by their corresponding PDB codes followed by the ligand code.

A dataset of 21 proteins containing 57 sites for ligand types CIT, MTX, MK1 and PGA (Table 1-iii) was curated and all vs all comparisons were carried out. The sites were then clustered based on their extent of dissimilarities. As evident from Figure 3 illustrating the cluster tree, sites corresponding to each ligand type cluster separately forming distinct groups among them. This serves to demonstrate that similarities among sites for a given ligand type are high in different proteins and the similarity scores are also sufficient to discriminate between sites for other ligand types.

### Sensitivity analysis

#### Sensitivity with respect to random perturbation of positions of site points

**Figure 4:**
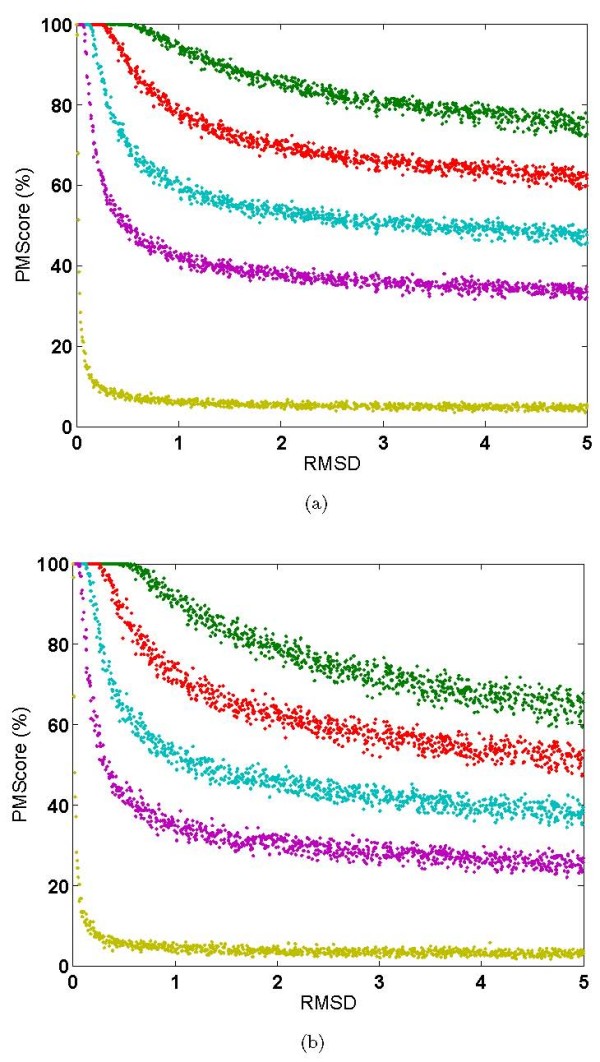
**Validation with respect to random perturbation of positions of site-points**. Random perturbations of site points for (a) a 54 atom ligand(PP8) and (b) a 34 atom ligand(MTX). PM*Scores *for perturbed sites with respect to its original site for different extents of perturbations(RMSD) are shown at different values of *τ *(1.0-green,0.5-red,0.25-cyan,0.125-blue,0.01-yellow).

The basic idea behind this validation is to understand the sensitivity of our algorithm, with respect to minor perturbations in the positions of binding site residues. This would encompass scenarios where the proteins being analyzed (a) have a minor error in the crystallographically determined coordinates, or (b) where the given site residue is flexible or (c) where the structure in hand is a homology model with minor changes in the site, such as due to mutations or binding of slightly different ligands. In all these cases the nature of the binding sites of the corresponding proteins is essentially the same and should be identified as similar by any site comparison algorithm. We chose two ligands of different sizes: phyenylpropanoic acid methyl ester(PP8) consisting of 54 atoms and methotrexate(MTX) consisting of 34 atoms. We perturbed the sites to varying extents within bounds of 0 to 5Å about 1000 times for each site individually. We then computed PM*Scores *for the original and the perturbed pair in each instance. A plot of PM*Score *vs RMSD between the sites is shown in Figures 4(a), 4(b) for the two ligands. Four different scoring schemes were used which differed in the *τ *parameter. While testing for sensitivity, this analysis also tests for an appropriate definition of similarity.

For a small perturbation of say < 2.0Å the PM*Scores *between the original and the perturbed site was seen to be very high with all scoring schemes for both the ligands. This indicates that the algorithm is robust enough to recognize two sites as similar even when minor perturbations to the extent of 2Å are present. When the perturbation was increased to 5Å the PM*Scores *showed lower values as expected. With scoring scheme-1(green, *τ *= 1.0) which was most liberal in terms of its similarity definition, the PM*Scores *varied very little even with high perturbation where as when scheme-5(yellow, *τ *= 0.01) was used, PM*Scores *moved to near zero even for small perturbations of about 0.1Å. The 2^*nd*^, 3^*rd *^and 4^*th *^scoring schemes corresponding to *τ *of 0.5, 0.25 and 0.125 respectively, showed in-between trends with a reasonable balance between sensitivity and robustness. The figure shows that the trend followed by the various schemes is consistent for different sizes of ligands. Given that the average coordinate error in crystal structures is in the order 0.5Å we have used the scoring scheme-2(red) as the default scheme for large scale analysis. *τ *is however implemented as a user defined parameter. This analysis indicates that the scores obtained are (a) reflective of the extent of similarities, (b) resistant to minor perturbations in the site, (c) scoring schemes are self consistent validating the basic logic used in the algorithm and (d) perturbed sites where some atoms have moved even up to 5Å are recognizable as similar to their original sites, albeit with lower PM*Scores*, because of retaining the overall nature of the site and high similarities with respect to remaining atoms in the site.

#### Sensitivity with respect to random perturbation of residue types of site points

**Figure 5:**
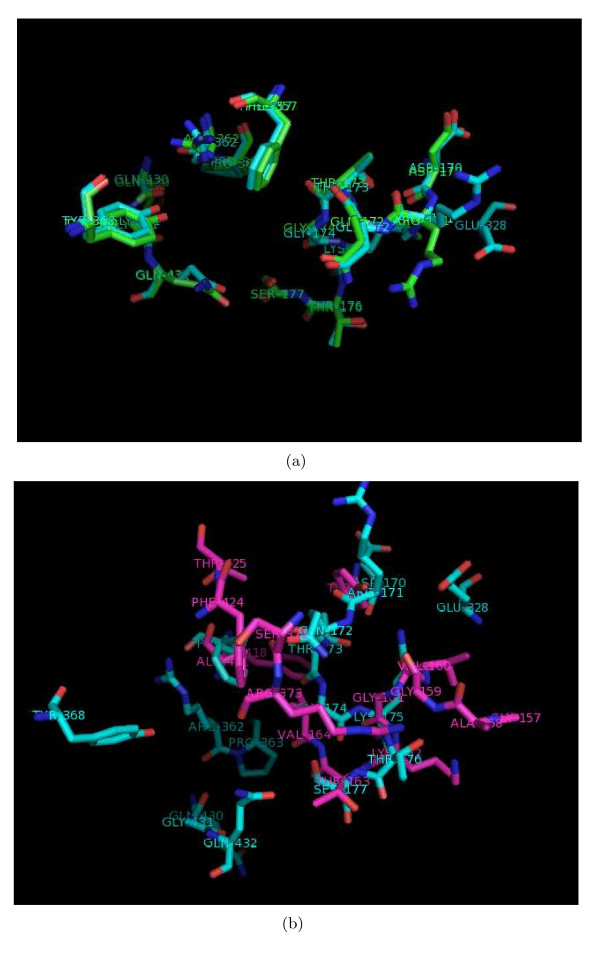
**Superposition of two ATP/ADP sites**. Examples illustrating validation of *PocketMatch*: Superposition of sites with (a) High PM*Scores *(80.9% for 1H8H-ATP and 1W0K-ADP) and (b) low PM*Scores *(25.8% for 1H8H-ATP and 1H8H-ADP).

**Figure 6:**
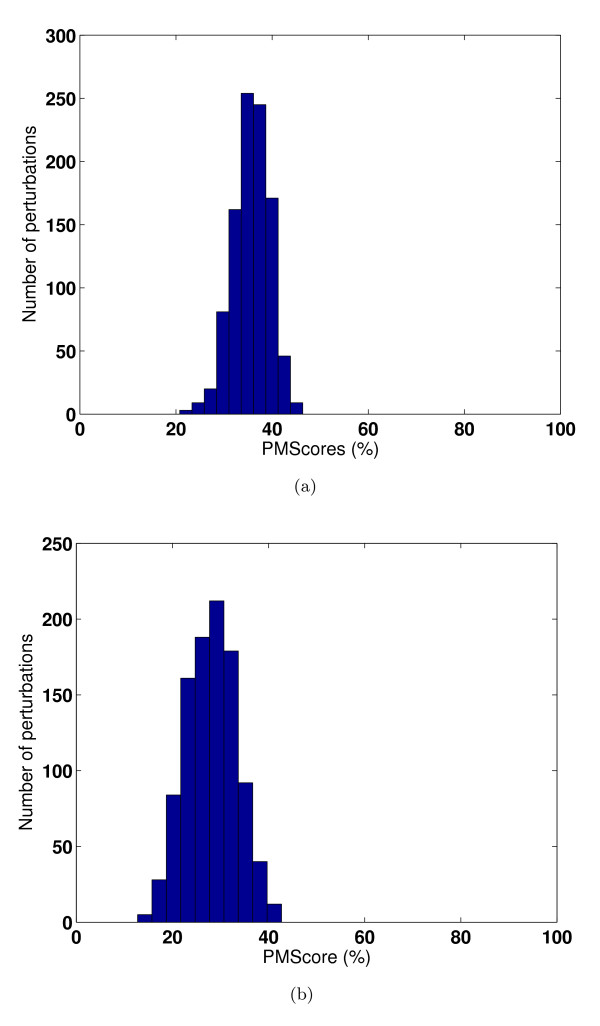
**Validation with respect to random perturbation of types of site-residues**. Perturbation analysis for the examples shown in Figure 5(a) for a pair of high scoring sites and 5(b) for a pair of low scoring sites. Distribution of PM*Scores *for 1000 randomly perturbed sites with respect to their original site is shown in both cases.

Given that spatial arrangement of specific amino acid residues at a given binding site dictates its recognition properties, we felt it was important to test the sensitivity of our algorithm to perturbations in the nature of the residues in the binding site without disturbing their spatial arrangement. We carried out this analysis on a pair of sites which were known to be similar to each other and another pair of sites which were known to be dissimilar to each other. To minimize site-specific biases that may arise during comparison, we chose one protein to be common between the two sites and all three to be nucleotide binding sites. One of the sites was kept constant while the other was perturbed. Of a possible 5^*N *^perturbations for a site a site of *N *residues, 1000 random perturbations were carried out and computed the PM*Score *of each of the perturbed sites with respect to the original site. The PM*Scores *for the unperturbed sites of the two chosen pairs (1H8H-ATP and 1W0K-ADP) and (1H8H-ATP and 1H8H-ADP) were 80.9% and 25.8% respectively whose superpositions are shown in Figures 5(a) and 5(b). The distribution of new PM*Scores *over the set of perturbed sites with respect to the original site is shown in Figure 6(a) for the first pair and Figure 6(b) for the second pair. Figure 6(a) shows that when perturbed, the similarity between two sites disappears and the scores get poorer. Figure 6(b) shows a similar trend indicating no high similarities were seen during random perturbations. Both these suggest that the arrangement of the site is specific and has been derived for a purpose and not just by chance. Our algorithm is sufficiently sensitive to detect changes in the nature of residues at the binding site. Our current implementation considers 20 amino acids classified into 5 groups and any change within the same group will obviously not be detected here. However the classification type is also implemented as a user defined parameter so as to consider each amino acid as a separate group to overcome this loss of sensitivity where more stringent analysis is required.

### Comparison of PocketMatch with other site matching methods

A benchmarking exercise of PocketMatch vis-a-vis the established binding site matching algorithms. SitesBase [14], SuMo [19], ProFunc [20] and a spherical harmonics method [16], was carried out, to place PocketMatch in context of other methods previously reported in literature.

*SitesBase *utilizes geometric hashing that works by evaluating many superpositions of triads of atoms between two sites. All atoms within 5 Å are considered to constitute a binding site. An atom-atom score is computed based on number of matching atoms in the sense of least RMSD. Empirically determined low and high scores are 25 and 40 respectively. Each score is flagged by Poisson-Index (PI) a measure of indicating the possibility of reaching that score by chance. The reported time for comparison of 82-atom pocket against a database of 33168 sites is 20 minute [14]. *SitesBase *is available on the web [21] as a database search engine with pre-defined proteins and sites, but not available for comparing user-defined structures. Hence some randomly chosen examples that were already available in the pre-computed database of SitesBase were considered for comparison with PocketMatch. The binding site search utility in *ProFunc *[20], a web service  for comprehensive analysis of proteins, employs the algorithm – JESS [22]. Jess uses a backtracking and depth first search method that works by determining appropriate correspondence between atoms in the pair of sites based on distance constraints. Ligand binding site templates are generated as triads of residues within 5Å each and having at least one non-hydrophobic residue flanking the location of a ligand in a PDB structure. Two sites are evaluated for similarity based on superposition (RMSD) and number of matches. A pure shape based method by [16] is based on representation of distribution of points from the centre of binding site by real spherical harmonic expansion coefficients. Binding sites are determined by SURFNET [23] as a collection of spheres filling the cavity. A ball of 1.4Å is then rotated around the collection to generate surface points. It is the shape of these points as a whole that is captured by the spherical harmonic coefficients and compared against other sets of points and hence other sites. The algorithm is not automatically available on the web or for local installation. Hence a dataset of 40 proteins reported by the authors was considered for comparison with PocketMatch. *SuMo *[19] is based on identification of common subgraphs between pairs of sites by greedy algorithmic strategy which may not lead to maximal common subgraph always. The nodes in the graphs are triangles where each triangle corresponds to a functional group in the binding site. Between a pair of sites, the triangles are matched followed by actual superposition of atoms of the sites by least squares optimization. The software is available at . The implementation of this method on the web allows comparison only with their pre-computed database of proteins and sites, but not available for comparing user-defined structures. In addition to these the align module of PyMol has also been used as another method of matching sites. PyMol considers sequence similarity and uses a least squares fit of the two sites, and reports number of residues matched and an RMSD for the matched residues.

**Figure 7:**
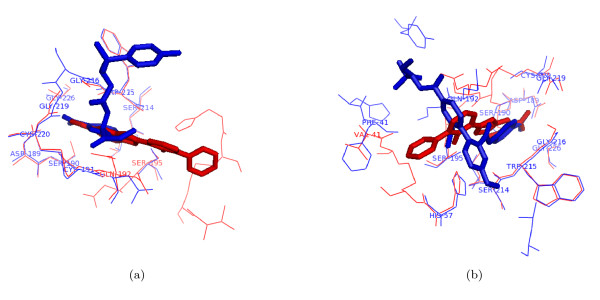
**Detection of part-similarities by PocketMatch**. Examples illustrating binding of different ligands in essentially the same binding pocket, but with different orientations. The part-similarities in these were identified correctly by PocketMatch. Binding of different trypsin inhibitors (stick models) complexed to trypsin variants (wire) as in PDB entries (a) 1GJC and 1V2Q and (b) 1GJC and 2AYW.

As evident from the above descriptions of the various methods, the binding sites are represented in different ways by different methods. The matching algorithms also vary significantly among them. Table 2 indicates the scores identified by the different methods for several protein-pairs belonging the same SCOP superfamily. While the obvious matches with high similarities are detected well by all methods, detection of matches with medium similarity is not so consistent. For example, the sialic acid binding site in 1A4G and 1NSC, both influenza virus neuraminidases bound to different ligands are identified by all methods with high scores. On the other hand, different methods fare differently with a hydrolase involved in blood clotting bound to different ligands. The ligands 2-(2-hydroxy-biphenyl)-1h-benzoimidazole-5-carboxamidine as in 1GJC, 2–5-[amino(iminio)methyl]-1h-benzimidazol-2-yl-6-(cyclopentyloxy)benzenolate as in 1O3P, 2-[2-([4-(diaminomethyl)phenyl]aminocarbonyl)-6-methoxypyridin-3-yl]-5-[(1-formyl-2,2-dimethylpropyl)amino] carbonylbenzoic acid as in 2AYW and Methyl n-[(4-methylphenyl)sulfonyl]glycyl-3-[amino(imino)methyl]-d-phenylalaninate [Nalpha-(2-naphthylsulfonylglycyl)-3-amidino-d,l-phenylalanine-isopropylester] as in 1V2Q. The different ligands bind to the same protein and essentially at the same binding site but with different orientations involving many common residues but some different amino acid residues. PocketMatch identifies all these pairs as significant matches with a PMScore of 50 to 88%, as shown in Table 2 and Figure 7(a),7(b). Some pairs were not detected by other methods whereas some were not available in their pre-computed lists. Some examples of protein pairs containing different folds but similar binding sites, reported in the literature were also chosen as shown in Table 2. PocketMatch was successful in detecting similarities in these pairs. To reflect part similarities that exist in them, PM*Score*_*min *_is also listed for these. Scores obtained from other methods are also listed. The performance as expected, is not consistent across all algorithms. For example, PyMol failed to align heme sites in Cytochrome c4 (1M6Z) and lignin peroxidase (1LGA), although PocketMatch found a significant similarity of more than 63% between them. None of the other methods detected this pair. SitesBase detected some of these pairs, but SuMo and ProFunc did not list them as possible matches.

**Table 2:** Comparison of PocketMatch with other site matching algorithms- ProFunc, SuMo, SitesBase and PyMol

**PDB1_LIG1**	**PDB2_LIG2**	**ProFunc**		**SuMo**	**SitesBase**		**PocketMatch**		**PyMol**	
				
		**Score**	**E- value**		**Score/Max.score = Ratio**	**PI**	**PMScore_Min_**	**PMScore**	**#R**	**rmsd**
**Pairs of proteins belonging to same SCOP families**										

1DHJ_MTX	4DFR_MTX	112	0.745	100	65/78 = 83.33	0	85.25	85.25	13,14,14	0.231

1A4G_ZMR	1NSC_SIA	112	0.277	76	74/79 = 93.67	0	99.91	88.39	16,16,17	0.141

1SDU_MK1	1SDT_MK1	X	-	NA	111/111 = 100	0	98.93	91.23	25,25,26	0.298

1B42_SAH	2VP3_SAH	241	9.8E- 4	91	100/130 = 76.92	0	99.4	89.29	19,20,19	0.088

1GJC_130	1V2Q_ANH	229	0.018	89	52/177 = 29.38	3E- 28	93.65	50.17	12,17,13	0.294

	2AYW_ONO	217	6.66E- 04	40	NA	-	56.9	52.29	15,17,17	1.186

	1O3P_655	X	-	NA	97/177 = 54.80	0	100	88.01	16,17,16	0.113

1ADD_1DA	2ADA_HPR	150	0.151	45	67/72 = 93.06	0	94.72	83.59	14,16,17	0.149

1KV5_PGA	2JGQ_PO4	104.586	23.327	X	NA	-	80.48	28.40	6,13,8	3.527

1BZC_TPI	1GFY_COL	174	0.176	27	57/95 = 60	2.4E- 40	96.87	75.41	12,15,13	0.236

1DJX_I3P	1DJY_I2P	X	-	58	46/53 = 86.79	2.5E- 38	100	69.05	10,10,12	0.160

1AJ6_NOV	1EI1_ANP	102	0.620	X	30/54 = 55.55	7.6E009	91.53	21.16	6,11,24	3.019

**Pairs of proteins belonging to different SCOP families**										

1ECM_TSA	4CSM_TSA	X	-	X	47/86 = 54.65	8.6E- 27	74.22	55.56	10,15,13	0.640

1M6Z_HEC	1LGA_HEM	X	-	X	X	-	67.58	63.85	12,23,24	5.875

1ZID_ZID	2CIG_1DG	X	-	NA	NA	-	58.94	56.01	5,27,29	5.691

1V07_HEM	1HBI_HEM	X	-	X	44/94 = 46.81	2.6E- 16	68.94	61.42	7,18,17	0.690

PMScore and PM*Score*_*min *_refer to PocketMatch scores with the two metrics described in the text. Scores for ProFunc, SuMo and SitesBase are as obtained individually from their respective web servers. Significance values where available are also indicated. SitesBase scores refer to number of matches (atoms)/maximum number of matches possible (total number of atoms present) = Percentage of atoms matched. PyMol (#R) refers to number of residues matched, number of residues in Site1, number of residues in Site2. X refers to cases where similarities are not detected with default settings of the algorithm, while NA indicates non availability that PDB entry in that dataset.

Isoniazid, a front-line drug used in the treatment of tuberculosis, is a pro-drug after activation, has been well known to target the inhA-encoded enoyl-ACP reductase (1ZID), thus inhibiting mycolic acid biosynthesis in *Mycobacterium tuberculosis*. Recently, a surprising observation of binding of isoniazid-adduct to dihydrofolate reductase has been reported [24] (PDB:2CIG). PocketMatch identified the isoniazid-adduct pockets in the two sites to be similar with a score of 56%, while the other methods failed to identify significant similarities between the two proteins.

It is no surprise that methods vary in their performance especially when the two sites are not highly similar, since different representations capture different aspects of the binding site. PocketMatch using a frame invariant sorted string of distances between point pairs captures the site by utilizing all available points in the site and hence reflects the shape and size of the site efficiently at the same time also reflecting the nature of amino acids, leading it to perform with higher sensitivity in detecting binding site similarities when some elements in the sites are different.

**Figure 8:**
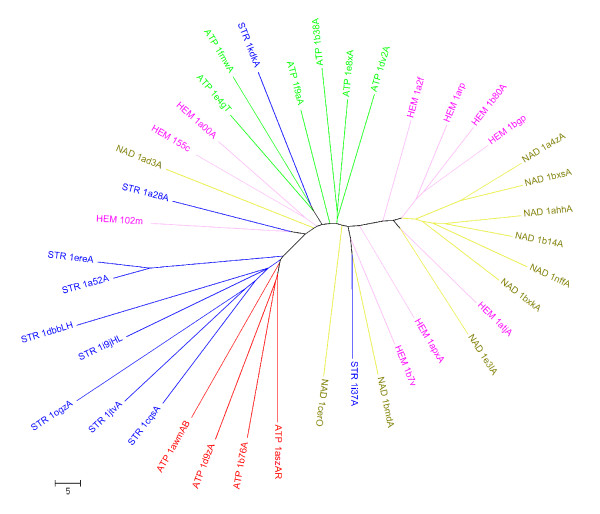
**A cluster tree of the 40 binding sites using the dataset of **[16]. The PDB codes are prefixed by ligand codes-STR indicating a steroid, ATP, NAD and HEM referring to heme. The chain IDS where appropriate are suffixed to the PDB codes. The proteins are coloured based on their ligand types. ATP sites are shown in two colours to reflect the two different types of sites.

A comparison of PocketMatch with the spherical harmonics based algorithm was also carried out, by using the dataset of 40 proteins reported by the authors. These proteins contained four different ligand types which are Steroid (STR), ATP, NAD and heme (HEM). Thornton and co-workers [16] reported a dendrogram of these 40 proteins based on their site comparison algorithm. Using the same dataset, we carried out an all-vs-all comparison of the sites and obtained PM*Scores*. We then clustered the proteins based on similarities in PM*Score *values. We observe that the cluster tree obtained by us is largely similar to that reported by [16], with major clusters correlating with individual ligand types Figure 8. 9 of the 11 structures with a steroid site, cluster together while the ATP sites cluster into two neat branches. The heme(HEM) and the NAD sites too largely form separate clusters. PocketMatch in fact appears to have fared better in clusters of ATP, NAD and heme. For example ATP_1b38A and Heme_1arp appear in one group by the spherical harmonics method but have grouped separately into clusters of ATP and heme respectively by PocketMatch (Figure 8). The proper grouping of ATP_1e8x and NAD_1ahh are other examples where PocketMatch has fared better. Although PocketMatch uses an entirely different, simpler representation scheme and matching algorithm as compared to that of [16], it is able to detect similar sites and discriminate against dis-similar sites in an efficient manner.

The benchmarking exercises have been useful in validating *PocketMatch *against those already available in the literature. Besides detecting obvious similarities, such comparison studies reveal interesting differences in the performance of different methods. Two major issues that feature in the comparisons are the sensitivity of similarity detection and speed. *PocketMatch *has an advantage in both these factors. The performance of *PocketMatch *was found to be good with different datasets used in this study. An added advantage with our method compared to others is that, scores reflecting local similarities PM*Score*_*min *_as well as global site similarities PM*Score *can both be obtained at the same time, thus readily yielding information about part similarities as well. This can be attributed in part to the fact that changes between two sites such as correlated mutations that maintain the same chemistry and distance in that pair are scored high by PocketMatch but not by some other methods. Similarly, amino acids at a binding site superposing well in structure but formed by dis-similar polypeptide segments also score high in PocketMatch.

### All vs all comparison – Site geometry vs residue types

**Figure 9:**
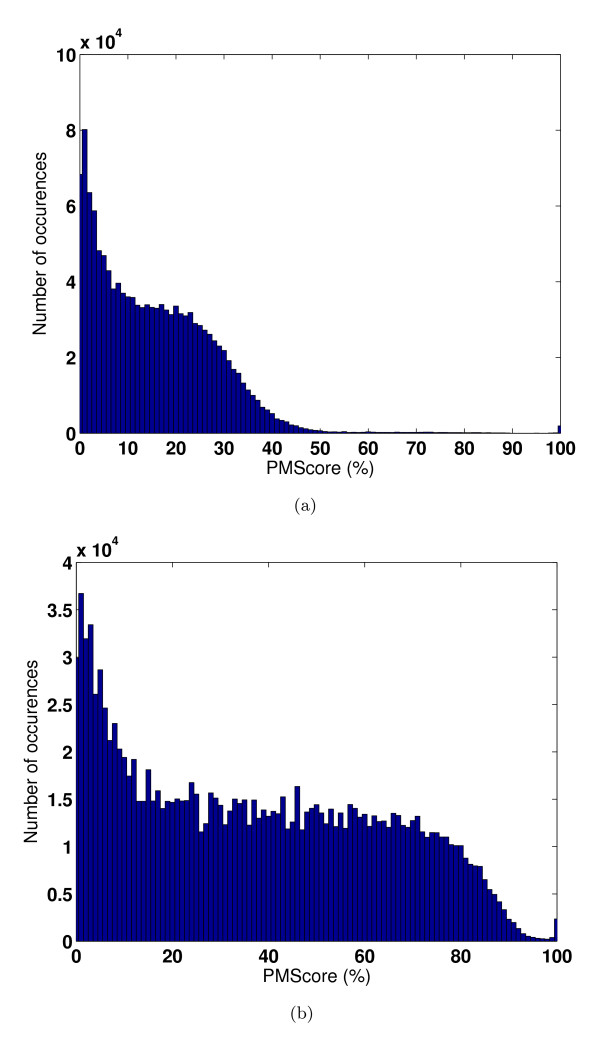
**Importance of residue group information over pure geometry**. All vs all comparison of 1146 sites of PDBBind dataset-ii (Table 1) using (a) default scoring scheme that uses both geometry and residue type information and (b) a scoring scheme that uses only site geometry. Histograms indicate distribution of number of pairs showing different PM*Score *values.

In order to estimate the sensitivity of our algorithm in terms of finding similarities for a given site in a large dataset, we performed an all vs all comparison in the PDBBind dataset Table 1-ii. The distribution of PM*Scores *across all pairs is shown in Figure 9(a). As evident from the histogram, majority of the pairwise scores are in the range of 0 to 40%. This means that any randomly chosen pair of two different sites will only have a score less than 40%. The histogram shows that only a small percentage of 0.8% of all possible pairs have scores higher than 50% which are indicative of true positives. Examination of 657231 such pairs indeed shows that they are true positives and in fact belong to the same SCOP class indicating that they are similar not only in their binding site architectures but also in their overall folds. This also shows that there are no false positives. It must be noted that this analysis was carried out with the scoring scheme, which we recommend as default that uses amino acid group information apart from the sorted distances. The same analysis was then repeated with another scoring scheme that differed from the previous one by not differentiating among amino acid types. Such a scoring scheme would identify similarities purely based on geometric features of the binding sites without considering their chemical nature explicitly. The distribution of PM*Scores *by this scheme is shown in Figure 9(b). Surprisingly, the same dataset showed a very different distribution of scores as compared to the previous analysis in Figure 9(a). Here a majority of pairs exhibited high scores indicating that many of the sites appear similar, obviously leading to a large number of false positives. However, when their chemical group information is added, differences between types of sites emerge. This analysis shows that shapes by themselves without considering chemical information is not sufficient to discriminate between different types of sites.

## Conclusion

A new algorithm has been developed to compare binding sites in accurate, efficient and high-throughput manner, where sites are represented as 90 lists of sorted distances flagged with residue type information. This representation therefore captures both the shape and chemical nature of amino acid types at the site. Extensive validation has been performed using different datasets. Sensitivity analysis has also been performed to analyze the performance of the algorithm with respect to perturbations of two types – (a) in the actual atomic positions of atoms of the site and (b) in the amino acid type at the site. Several scoring schemes have been analyzed by virtue of which a scoring function with a good balance between sensitivity and ability to detect similarities has been identified and recommended as the default scoring scheme. Perturbation studies where the geometry of a given site was retained but the residue types were changed randomly, indicated that chance similarities were virtually non-existent. Our analysis also suggests that shape information alone is insufficient to discriminate between diverse binding sites. However, combining shape information with chemical grouping of amino acids at the site enables discrimination between different types of sites.

## Methods

### Datasets

*PocketMatch *has been validated on a variety of datasets – PDBBind, a set of tetrameric proteins and a curated dataset containing known similarities of four ligand types (Table 1). To eliminate noise in the datasets, sites corresponding to *small ligands *with less than 6 non-hydrogen atoms and *covalently bound *ligands were not considered here. The PDBBind dataset [25] contains a comprehensive curated set of 1091 protein-ligand complexes determined crystallographically. Using this, two sub-datasets were derived – Table 1-i: A dataset which has only one ligand site for one ligand type in each protein, amounting to 786 proteins and 893 sites and Table 1-ii: A dataset in which ligands suggested to contribute to noise by Jackson and co-workers [14] were removed, but all sites for all ligand types were considered, amounting to 456 proteins and 1146 sites corresponding to 289 ligand types. Tetramers (Table 1-iv), obtained from PQS server, containing 3768 proteins has been curated to yield a dataset of 1525 proteins having 11301 sites has been chosen for studying the sensitivity of *PocketMatch *with respect to recognizing known highly similar sites. Another dataset representing multiple sites for four known ligands was curated (Table 1-iii). 51 sites from 27 different proteins in PDBBind for Citrate (CIT), Methotrexate (MTX), Indinavir (MK1) and phosphoglycerate (PGA) were chosen for the dataset. A distance metric measuring the dis-similarity between these sites was computed using *PocketMatch *and their clustering was studied.

### Scheme for Validation

We have validated the algorithm based on (a) SCOP equivalences between pairs of proteins at 4^*th *^level of SCOP [1], (b) similarities among multiple sites for a given ligand in the tetramer dataset Table 1-iv and (c) clusters formed by sites whose similarities have been previously identified by independent analysis. To compare with SCOP, we constructed a matrix, *M*_1 _where each of the rows and columns correspond to list proteins and each element $M_{1_{ij}}$ corresponds to a score assigned to that pair of proteins, i and j. A score of 1.0 was assigned to those pairs that had the same SCOP class. All other pairs were assigned a score of 0. Then we constructed another matrix, *M*_2 _of the same dimension where a score of 1.0 was assigned to those pairs whose top scoring site-pair had a PM*Score *greater than a threshold and score of 0 for all others. The two matrices were then compared using the XOR, a logical operator to determine the extent of agreement between the fold and the site metrics in finding similarities in all pairs of proteins represented in the matrices. Understandably, the same comparison can also be done through the XNOR operator, the complement of XOR, which will yield the same information.

#### Schemes for sensitivity analysis

Sensitivities of *PocketMatch *(a) with respect to geometry and (b) with respect to specificity of residue types at the site, were studied. For (a), we applied tiny perturbations to coordinates to each of the points representing sites, leading to generation of altered structures within bounded a RMSD and computed PM*Scores *between pairs of perturbed and reference sites. These plots are obtained for various sizes of binding sites. For (b), we randomly assigned residue group information to each of site points and computed PM*Scores*.

#### Method for random perturbation to positions of site-points

Our method for random perturbation applies random displacement from a uniform distribution to each of the points *C*_*α*_, *C*_*β*_, and *C*_*centroid *_corresponding to each residue as described in (*Sub-routine 2*).

*Sub-routine *2 Performing random perturbation to positions of points of the site

a Let the 'net required RMSD' be *ρ *and number of intermediate randomly perturbed point-sets be *K*.

b In order to generate, *K *perturbed sets of points,

   **for **EACH *δ *= 0 till *ρ *in steps of $\frac{\rho }{K}$; **do**

      - Perturb the actual set of points to generate a new point-set at an RMSD of *δ*.

      - Let each point be represented by $\bar{x}$ = (*x*_1_, *x*_2_, *x*_3_) and let the random vector be $\bar{r}$ = (*r*_1_, *r*_2_, *r*_3_).

      - Initialize each *r*_*i *_to $\frac{\cos(2.0*\pi *random())}{1+RAND\_MAX}$; where random() function generates a random number between 0 and *RAND_MAX *from 'uniform distribution'.

      - Normalise $\bar{r}$ by reinitializing each $r_{i}=\frac{r_{i}}{\sqrt{\sum_{i=1}^{3}r_{i}^{2}}}$.

      - Apply the present displacement, $\bar{r}$ * *δ *to each atom which set each ${\bar{x}}^{new}={\bar{x}}^{old}+\bar{r}*\delta$

      - These steps generate a perturbed set of points with a net RMSD of *δ*.

   **end for**

c For each of the perturbed point-set generated, compute RMSD with respect to the unperturbed version and measure PM*Scores*.

### Method for random perturbation of types of site-residues

Our method for random perturbation of group numbers assigned to residues of site involves assignment of random integers between 0 and 4 from a 'uniform' distribution to the residues represented by three points, *C*_*α*_, *C*_*β*_, and *C*_*centroid *_as described in (*Sub-routine 3*).

*Sub-routine *3 Random perturbation of types of site residues

a Get *C*_*α*_'s alone for a given site represented in *C*_*α*_, *C*_*β*_, and *C*_*centroid *_– format.

b To each of the *C*_*α*_'s assign a random number between 0 and 4 using $\frac{5*random()}{\left(RAND\_MAX+1.0\right)}$

c Copy back the modified group information of *C*_*α *_atoms back to the file respresenting site by *C*_*α*_, *C*_*β*_, and *C*_*centroid *_points.

d The PM*Score *is then computed for the perturbed site with respect to unperturbed version.

#### Implementation

The software *PocketMatch *was developed on *gcc (GCC) 3.4.3 20041212version 4.1.2 *on Linux 2.6.9-5.ELsmp machine. A parallelized version of the software using MPI-C libraries was also developed and implemented both on a standard Quad-core machine (Intel (R) Core (TM) 2 Quad CPU @ 2.4 GHz; Address space: 32 bits physical, 48 bits virtual; 4GB RAM) and also on a IBM BlueGene cluster and tested with 512 processors. *Matlab *version 7.1 from Mathworks  was used for generating plots and histograms. Cluster tree was constructed using neighbour-joining method of *phylip-3.67 * and viewed using *PhyloDraw *[26]. *PyMol * version 0.99rc1 was used for visualizing various structures.

## Availability

The software can be accessed at 

## Authors' contributions

YK, a graduate student developed and implemented the method under the guidance of his advisor NSC. Both authors discussed and wrote the paper.
